# A Gold Nanoparticles Enhanced Surface Plasmon Resonance Immunosensor for Highly Sensitive Detection of Ischemia-Modified Albumin

**DOI:** 10.3390/s131012794

**Published:** 2013-09-25

**Authors:** Guang Li, Xian Li, Meng Yang, Meng-Meng Chen, Long-Cong Chen, Xing-Liang Xiong

**Affiliations:** 1 Laboratory of Biomedical Engineering, Chongqing Medical University, 1 Yixueyuan Road, Chongqing 400016, China; E-Mails: aza-lg@163.com (G.L.); yymm1219@163.com (M.Y.); cmm1029@163.com (M.-M.C.); 2 Key Laboratory of Biorheological Science and Technology, Ministry of Education, Bioengineering College, Chongqing University, 174 Shapingba Main Street, Chongqing 400044, China; E-Mail: lixian810@gmail.com

**Keywords:** ischemia modified albumin, surface plasmon resonance immunosensor, gold nanoparticles, signal enhancement

## Abstract

In this study a novel sensitive nanogold particle sensor enhancement based on mixed self-assembled monolayers was explored and used to construct a Surface Plasmon Resonance (SPR) immunosensor to detect Ischemia Modified Albumin (IMA). Compared with a direct binding SPR assay at a limit of detection (LOD) of 100 ng/L, gold nanoparticles (AuNPs) of 10 nm dramatically improved the LOD of IMA to 10 ng/L. Meanwhile, no interfering substance that may lead to false positive results was identified. These results suggested that the SPR biosensor presented superior properties, and provided a simple label-free strategy to increase assay sensitivity for further acute coronary syndrome (ACS) diagnosis.

## 1. Introduction

With a sharp rise in the rate of occurrence of non-cardiac chest pain, identification of transient myocardial ischemia and angina has been one of the most important challenges in the clinic. Delayed diagnosis and therapy not only increase costs, but also lead to serious cardiac disease and mortality [1]. For the diagnosis of chest pain patients, only 50% of these patients manifest typical acute coronary syndrome (ACS), and both physical examination and electrocardiogram (ECG) ST-elevation can only provide poor specificity. What's more, in the absence of a “gold standard” assay for myocardial ischaemia, patients will undergo a series of expensive and frustrating non-specific tests. Myoglobin, creatine kinase-MB (CK-MB) and troponin have been used frequently as cardiac makers [2], but their use is limited by insufficient sensitivity for the ischemic condition in the initial onset period. These biochemical markers release from myocardial cells only after irreversible lesions [3], hence, it is impossible to detect the rising signals in the blood with transient and unconspicuous ischemia. Nevertheless, a biomarker that can reliably detect ischemia, regardless of whether necrosis has occurred, and before these biochemical markers are released, is sorely needed.

Recently, Ischemia Modified Albumin (IMA), approved by the United States Food and Drug Administration (FDA), has been reported to be the only marker capable of reflecting myocardial ischemia condition [4]. When exposed to ischemic conditions, the N-terminus of albumin is damaged or occupied by copper ions, which makes it unable to bind cobalt and other heavy metal ions [5]. The IMA concentration in the blood shows an initial up-regulation within a few minutes after the emergence of myocardial ischemia and eventually drops back to normal within 6–12 h. The case leads us to the conclusion that it can be implicated in the detection of myocardial ischemia prior to necrosis. As for IMA detection, Bar-Or [6] firstly established a method by spectrophotometry. At present, the IMA concentration can be determined using the Albumin Cobalt Binding (ACB) test. However, the test currently has the drawbacks of indirect detection, false positives, and it is unreliable when patients have a blood albumin level of less than 34 g/L [7]. Therefore, it is urgent to develop a method that could overcome these above flaws and measure IMA concentration directly.

Surface plasmon resonance (SPR) is an optical sensor based on the changes in the refractive index of material on the gold surface [8]. Compared with other techniques [9], SPR offers a series of advantages, including label-free assays, rapid-analysis and real-time include on-site or *in-situ* detection [10]. Account for these superior properties, efforts have been made toward SPR biosensors for specific biomolecular binding events, such as protein (enzymes, antigens and antibodies) interactions [11,12], nucleic acid-nucleic acid/protein hybridization [13,14], and virus recognition events [15]. Past studies have shown that current SPR methods can detect approximately 100 ng/L proteins [16], which sensitivity is sometimes insufficient for measuring samples. During recent years, colloidal gold nanoparticles have been widely used as a label for analytical signal amplification [17,18]. They possess advantages such as narrow size distribution, rapid and easy synthesis, efficient surface modification, and desirable biocompatibility [19].

In this study, our approach to development of a new kind of SPR biosensor for the determination of IMA is essentially based on assembling anti-IMA onto an AuNP-modified gold chip. The microfluidic-integrated system has been developed to transport a mass of molecules over the chip surface, which exhibits excellent ability to improve the detection limit compared with conventional label-free SPR analysis.

## 2. Experimental Section

### 2.1. Reagents and Apparatus

N-hydroxysuccinimide (NHS), 1-ethyl-3-(3-dimethylaminopropy) carbodiimide hydrochloride (EDC), 11-mercaptoundecanol (MUOH), 11-mercaptoundecanoic acid (MUA), 16-mercaptohexadecanoic acid (MHA), nanogold (10 nm), were obtained from Sigma-Aldrich (St. Louis, MO, USA). Ischemia Modified Albumin (IMA) was bought from Sichuan Xincheng Biological Technology Co., Ltd. (Chengdu, China). Ischemia modified albumin antibody (anti-IMA) was purchased from Shanghai Gaochuang Chemical Technology Co., Ltd. (Shanghai, China). Ethanolamine (MEA, 1 mol/L, pH = 8.5), phosphate buffer solution (PBS, 0.1 mol/L, pH = 7.4) and other reagents were all of analytical reagent grade. Response curves were aquired on a HPSPR-6000 Bioanalyzer (Xunjie, Henan, China). The sensor surface was characterized by AFM using a SPI3800N-SPA400 system (Seiko, Japan).

### 2.2. Cleaning of the Chip

The gold sides were cleaned with freshly prepared piranha solution (70% H_2_SO_4_, 30% H_2_O_2_) at 50 °C for 10 min to remove all organic contaminants, and then rinsed with copious amount of ultrapure water, and dried under a stream of nitrogen.

### 2.3. Immobilization of Anti-IMA

First, the gold side was immersed in 0.01 mol/L MUA at room temperature for 24 h to form a self-assembled monolayer (SAM). As to the mixed SAM (mSAM) surface, it was functionalized with a 1:9 (v/v) ratio of MHA and MUOH on the gold side. The chip was then washed with ethanol and ultrapure water, and dried under nitrogen. These steps provided stable and fully covered SAMs on the surface. Subsequently, the gold side was immersed in a solution of 0.1 mol/L NHS and 0.4 mol/L EDC with 1:1 (v/v) ratio for 30 min. Anti-IMA was then immobilized onto the surface with different concentration in PBS (pH = 7.4) for 1 h. At last, the chip was immersed into a solution of 1 mol/L ethanolamine (pH = 8.5) for 20 min to inactive the unreacted esters.

### 2.4. Preparation of IMA-AuNP Complex

The AuNPs were dispersed in 0.1 mol/L K_2_CO_3_ solution (pH = 9), and then incubated with IMA overnight at 4 °C. The complex was centrifuged at 9000 r/min for 20 min to remove the supernatant, the left was then dispersed in PBS buffer and stored at 4 °C [20].

### 2.5. SPR Assays for IMA

**Figure 1.**
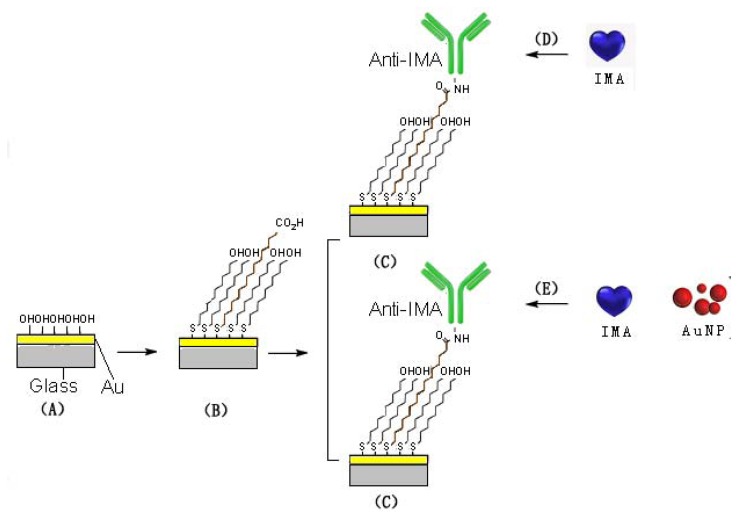
Sequential assay format: (**A**) a clean chip (**B**) a mSAM gold surface, (**C**) an Anti-IMA immobilized sensor surface, (**D**) IMA binding on the sensor surface(Format A), (**E**) IMA/AuNP binding on the sensor surface(Format B): both (D and E) return to (C) after surface regeneration.

A series of IMA solutions (0–100 μg/L) in PBS buffer was firstly applied to the chip immobilized with Anti-IMA for 10 min at a rate of 20 μL/min. The unbound molecules were then washed away by PBS. Then, the chip was regenerated by injecting mixture of 0.1 mol/L NaOH and 0.05% SDS for 8 min each time, with another concentration of IMA inserted (Format A). Signal enhancement by IMA/nanogold (10 nm) was also performed in a sequential injection procedure similar to the steps above (Format B). The entire process was shown in Figure 1.

## 3. Results and Discussion

### 3.1. Evaluation of mSAMs on the SPR Immunosensor Signal

**Figure 2.**
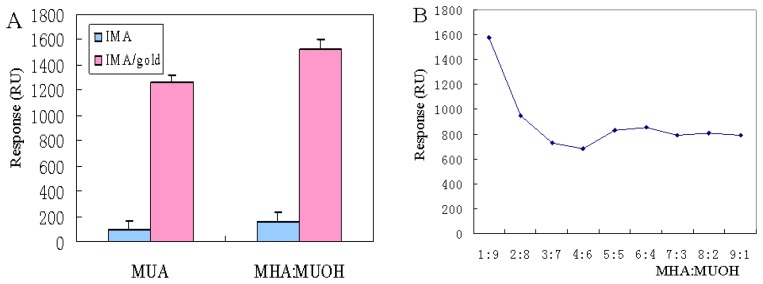
Effects of MUA and MHA/MUOH mixed self-assembled chips on surface for Format A (IMA 100 μg/L) and B (IMA-AuNP complex, 100 μg/L IMA, AuNP size 10 nm) (**A**) and the changing proportion of MHA:MUOH from 1:9, 2:8, 3:7, 4:6, 5:5, 6:4, 7:3, 8:2 to 9:1. (**B**) The experiments of graphic (B) were achieved employing Format B.

SPR is an optical-electrical phenomenon based on the plasmon induced electronic field over the sensor surface. Studies had shown that the SPR response signal was affected by the distance from the gold sensing surface [21]. Any SPR response is closely related to the tiny distance between analyte and the surface. SPR immunoassays are currently performed using CM5 chips [22], where the 100 nm distance taken by a carboxymethylated dextran matrix on the chip significantly lengthens the response distance. Therefore, we attempted to seek a shorter binding layer such as SAM on a bare gold chip. In this study, MUA and a mixture of MHA and MUOH were examined as a SAM on the chip surface. The formation of monolayer with the former resulted in relatively minor adsorption compared with the latter (Figure 2A). The results clearly demonstrated that the mSAM surface achieved higher signal enhancement than the CM5 surface.

Thus, we chose the mixture as the replacement for carboxymethylated dextran, whereas the optimized ratio of mSAM was still under discussion. Different mSAMs were formed by immersing chips into solutions containing MHA and MUOH at a ratio of 1:9, 2:8, 3:7, 4:6, 5:5, 6:4, 7:3, 8:2, 9:1. Results indicated that when the ratio was 1:9, the binding exhibited the highest SPR responses (Figure 2B). Hydroxyl-terminated SAMs (MUOH) decreased non-specific binding and were utilized as spacers to form the chip surface, while carboxyl-terminated SAMs (MHA) were exploited as functional linkers to immobilize IMA for immunoassays. Because of steric hindrance, bulk massed carboxyl groups showed reduced reactivity compared with the small sized hydroxyl groups. Choi [23] reported that two heterogeneous chain lengths resulted in less steric hindrance. Tamada [24] also demonstrated that longer carbon chains produced rougher surfaces than shorter chains. As the carbonchain length increased, a phase segregation occurred from order to disorder. A rough surface was formed through van der Waals attraction between the chains and therefore raised the surface reactivity.

### 3.2. Optimization Conditions for the AuNP

**Figure 3.**
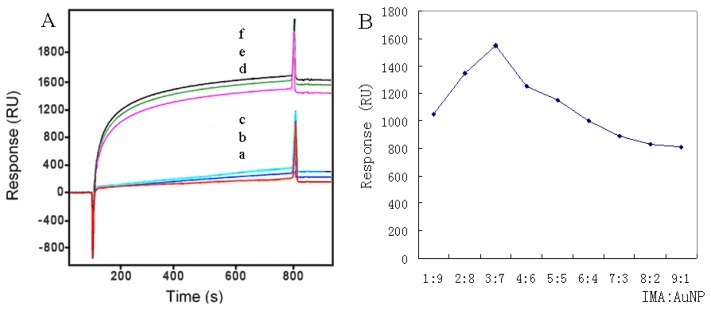
Sensograms showing the bioconjugation yield with different strategies (**A**). IMA concentration of 100 μg/L with three replications from a to c (Format A) and similarly, AuNP enhancement achieved employing IMA from d to f (Format B). The effect of the IMA:AuNP ratios (1:9, 2:8, 3:7, 4:6, 5:5, 6:4, 7:3, 8:2, 9:1) achieved on SPR (**B**), the experiments of graphic (B) were developed with IMA-AuNP (100 μg/L IMA, AuNP size 10 nm) of the different dilution ratios of AuNP (Format B).

According to Figure 3A, the use of AuNP produced a significant increase of the signal in comparison to the signal achieved without any enhancement. Other authors [25–27] reported that different sized AuNP could produce various levels of signal enhancement. While the recognition sites on the antibody were limited, and small sized AuNP possessed the advantage of better diffusion to the surface. In this study, we chose 10 nm AuNP, and the achieved signal was about 10 times higher in respect to the primary antibody signal.

The effect of different ratios of IMA:AuNP on signal enhancement was also investigated. Signal enhancement depended crucially on the particle size which was composed of AuNP and surrounding IMA molecules. Appropriate ratios might increase the chances of the particles binding to the surface. As shown in Figure 3B, the optimal here was chosen to be 3:7 (v/v).

**Figure 4.**
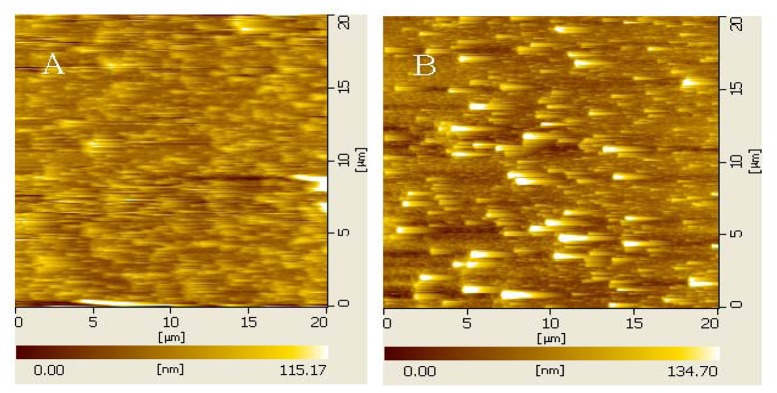
AFM images of silicon chip substrate modified with IMA at concentration of 25 μg/L (**A**) and IMA–AuNP complex of 25 μg/L (**B**).

To account for the large size of AuNP, the IMA-AuNP complex binding on the surface was characterized by atomic force microscopy. The AFM images shown in Figure 4 were obtained in the contact mode with a scan rate of 1.0 Hz. The complex was observed to combine uniformly with its antigen, suggesting that the IMA-AuNP complex had excellent dispensability in buffer solution, as well as high specificity against the antigen.

### 3.3. Effect of SPR Detectability

**Figure 5.**
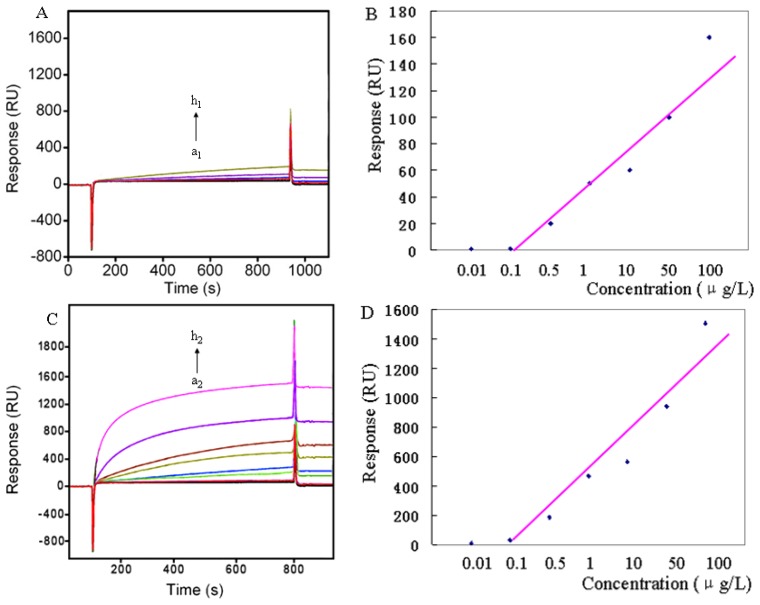
The sensorgrams obtained with IMA concentrations of 0 μg/L, 0.01 μg/L, 0.1 μg/L, 0.5 μg/L, 1 μg/L, 10 μg/L, 50 μg/L, 100 μg/L, (from a to h), (**A**), direct assay: IMA only (20 L/min, 3 min); (**C**), AuNP enhanced assay: IMA/AuNP (10 nm) enhancement, IMA/AuNP:PBS (2.5:1, v/v), 10 L/min, 4 min. Calibration curve of ΔRU *versus* IMA concentration with Format A (**B**), Format B (**D**). Some error bars were too small to be seen (based on two measurements for each IMA concentration).

In the SPR sensing system, IMA firstly bound to the anti-IMA immobilized on the chip surface (Format A), then the mixture IMA-AuNP was inserted (Format B). In order to clarify the analytical performance of the biosensor proposed in this work, experiments were carried out with series of IMA concentrations. Significant signals were obtained with inserting in two formats and their signal values were also well consistent with the enhancement theory at high and low concentration. In Figure 5, the response signal increased with the increase of IMA concentration. Moreover, the time required for binding was only 4 min, with this procedure no labeling is required and the target can be detected in real-time. The two formats (A, direct assay; B, AuNP enhanced assay) were also evaluated (Figure 5B,D).

For direct assay, the sensor demonstrated a LOD of 100 ng/L. On the surface-coated Anti-IMA, the binding performance indicated a 9.4-fold with AuNP enhancement assay. The signal enhancement by Format B lowered the Anti-IMA concentration from A 100 μg/L for the conventional assay to B 5 μg/L, which significantly improved the LOD from A 100 ± 18 ng/L to B 10 ± 1.7 ng/L (Table 1). The results are analyzed statistically using first order theory.

### 3.4. Selectivity of Developed IMA Biosensor

For the screening of ACS, the negative predictive value and the sensitivity of IMA were higher than that of conventional biochemical markers, while the specificity was lower, especially for interfering substances including heparin (Hep), hemoglobin (Hb), bilirubin (Bil) and triglyceride (TG). In our study, to examine whether IMA result differed significantly between the ACS group and non-ACS groups with pure IMA under certain concentration, we chose 198 cases confirmed as ACS; using the SPR assay, the sensor demonstrated almost the same response signal as those control groups (Table 2).

**Figure 6.**
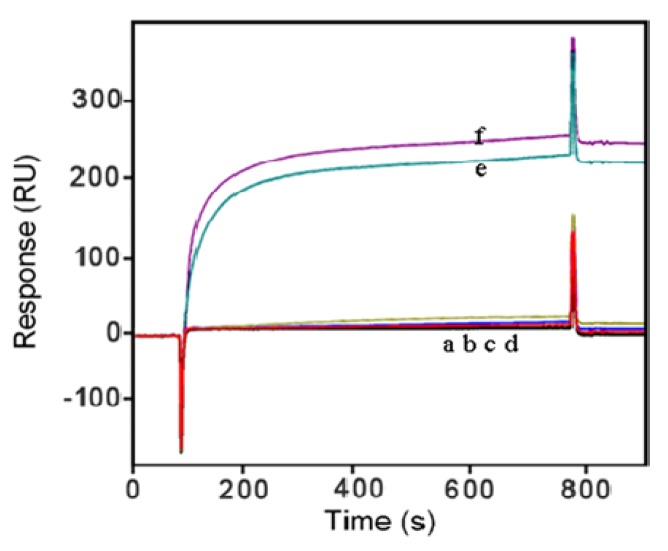
The sensor grams obtained with different analytes, Hep, Hb, Bil, TG (from a to d), IMA sample with ACS (e) and control group (f). The current of each interfering substance was obtained with 500 ng/L concentration in 0.1 M PBS solution (Format B).

Meanwhile, Hep solution (500 ng/L) in PBS buffer was prepared and passed over the Anti-IMA modified surface for 10 min, Hb, Bil and TG were also tested in a sequential injection procedure similar to Hep (Figure 6). The results showed that hardly any signal improvement was observed, that is to say, this approach has been proved to be reliable with its high selectivity.

## 4. Conclusions

To sum up, the study described above demonstrates the potential of an ultra-sensitive SPR assay for IMA by a mixed SAM configuration to reduce the 10 nm distance of AuNP on the sensor surface. The developed SPR biosensor works well, especially at low concentrations of IMA, and there is no interference that may lead to false positives. Here, IMA can be detected at 10 ng/L with AuNP, which is good news for patients with a blood albumin level of less than 34 g/L when ACB is unworkable. The modified biosensor displays excellent signal response, high sensitivity and much lower detection limits, which provides an effective approach for realizing a direct assay of IMA. Further study plans include applying this technique to other clinical analytes for further signal enhancement using other particles or more thoughtful conjunctions to improve the sensitivity. Undoubtedly, with the growing attention and advances of performance in SPR, the proposed technique holds great promise in clinical diagnosis.

## Figures and Tables

**Table 1.** Summary of IMA SPR assay parameters including ratios of signal enhancement and LOD improvement.

**Format**	**Anti-IMA** **(μg/L)**	**LOD** **(ng/L)**	**Signal** **Enhancement Ratio**	**LOD** **Improvement Ratio**
A	100	100 ± 18	n/a	n/a
B	5	10 ± 1.7	9.4	10

Note: All the parameters are expressed as the average and standard deviation (n = 3 channels).

**Table 2.** Selectivity of developed IMA biosensor.

**Interfering Substance**	**Concentration (ng/L)**	Δ**RU**	

		**av^2^**	**SD**
Hep	500	7.2	9.44
Hb	500	6.6	17.68
Bil	500	9.5	12.91
TG	500	8.4	15.73

IMA(ACS)	500	205.6	11.56

IMA(non-ACS)	500	227.3	13.21

Note: All the parameters are expressed as the average and standard deviation (n = 3 channels).
